# Managing the Uncertainty of “Precision” While Navigating Goals of Care: A Framework for Collaborative Interpretation of Complex Genomic Testing Results in Critically-Ill Neonates

**DOI:** 10.3390/children13010034

**Published:** 2025-12-26

**Authors:** DonnaMaria E. Cortezzo, Katharine Press Callahan, Bimal P. Chaudhari, Elliott M. Weiss, Monica Hsiung Wojcik, Krishna Acharya, Amy B. Schlegel, Kevin M. Sullivan, Jessica T. Fry

**Affiliations:** 1 Division of Neonatology, Connecticut Children’s, Hartford, CT 06106, USA; 2 Division of Pain and Palliative Care, Connecticut Children’s, Hartford, CT 06106, USA; 3 Fetal Care Center, Connecticut Children’s, Hartford, CT 06106, USA; 4 Department of Pediatrics, School of Medicine, University of Connecticut, Farmington, CT 06030, USA; 5 Division of Neonatology, The Children’s Hospital of Philadelphia, Philadelphia, PA 19104, USA; callahankp@chop.edu; 6 Department of Medical Ethics and Health Policy, The Perelman School of Medicine, University of Pennsylvania, Philadelphia, PA 19104, USA; 7 Division of Neonatology, Nationwide Children’s Hospital, Columbus, OH 43205, USA; bimal.chaudhari@nationwidechildrens.org (B.P.C.);; 8 Division of Genetic and Genomic Medicine, Nationwide Children’s Hospital, Columbus, OH 43205, USA; 9 The Steve and Cindy Rasmussen Institute for Genomic Medicine, Nationwide Children’s Hospital, Columbus, OH 43205, USA; 10 Department of Pediatrics, College of Medicine, The Ohio State University, Columbus, OH 43210, USA; 11 Treuman Katz Center for Pediatric Bioethics & Palliative Care, Seattle Children’s Research Institute, Seattle, WA 98101, USA; 12 Department of Pediatrics, School of Medicine, University of Washington, Seattle, WA 98101, USA; 13 Divisions of Newborn Medicine & Genetics and Genomics, Boston Children’s Hospital, Boston, MA 02115, USA; 14 Division of Neonatology, Children’s Hospital of Wisconsin, Milwaukee, WI 53226, USA; kkacharya@mcw.edu; 15 Division of Neonatology, Nemours Children’s Health, Wilmington, DE 19803, USA; 16 Department of Pediatrics, Sidney Kimmel Medical College, Thomas Jefferson University, Wilmington, DE 19803, USA; 17 Division of Neonatology, Ann & Robert H. Lurie Children’s Hospital of Chicago, Chicago, IL 60611, USA; jtfry@luriechildrens.org; 18 Department of Pediatrics, Feinberg School of Medicine, Northwestern University, Chicago, IL 60611, USA

**Keywords:** genomic testing, neonate, decision-making, diagnostic uncertainty, prognostic uncertainty, palliative care, complex care, navigating goals of care

## Abstract

Each year, many neonates are born with genetic diagnoses that carry a range of prognoses. As the types and availability of genetic testing have expanded, neonatal intensive care units (NICUs) have served as “launching points” for their clinical application. Broad genetic testing has both improved diagnostic precision and expanded uncertainty. Genetic information may be explicitly uncertain, as in the case of a variant of unknown significance (VUS). But it is also frequently uncertain whether/how the information relates to a patient’s phenotype or what it may mean for a child’s future. Even without ambiguity in the diagnosis or prognosis, the significance within a clinical and familial context may be less certain. Applying the information to clinical care is complex and may engender confusion among clinicians and families as readily as it offers guidance. Since genetic testing results can impact management and, at times, end-of-life decisions, misunderstanding and misapplication of genetic results pose a significant risk. We describe a hypothetical case of an infant with congenital hypotonia and respiratory failure. The family, after discussions with the care team about medically appropriate care paths, is navigating goals of care and considering tracheostomy placement for chronic mechanical ventilation. They consent to rapid genome sequencing in hopes of better understanding the etiology and severity of the neuromuscular condition. We explore three possible scenarios following different genomic results. With each, we discuss how the results may impact decision-making about the best plan of care. We propose a framework for navigating discussions about genetic testing results with families of critically ill children. We illustrate the importance of a multidisciplinary approach with collaboration between neonatology, genetics, and palliative care. By employing the strengths of each subspecialty, providers can manage the inherent uncertainty in genetic testing results, help determine the meaning of the results to the family in the context of their child’s medical care, and enhance the care and support of critically ill neonates and their families.

## 1. Introduction

As the types and availability of genetic testing have expanded, neonatal intensive care units (NICUs) have served as “launching points” for their clinical application. This is driven by multiple factors: the high prevalence of genetic disease in critically ill newborns, a desire for more precise prognostic information, the ability to provide diagnostic information of clinical utility, and the perceived need for urgent decisions regarding life-sustaining therapies [1,2,3,4,5,6,7,8,9,10,11,12,13,14,15,16,17,18]. Genetic tests are returning faster, which means results can increasingly be integrated into acute care decisions [1]. While this can provide timely and clinically relevant information to aid in the management of neonates, new genetic technologies, such as exome and genome sequencing, may also generate abundant and, at times, uncertain information [19]. For instance, testing may reveal a variant of unknown significance (VUS), a poorly categorized diagnosis, or a diagnosis with a range of potential outcomes [20,21]. In other words, at times, genetic information may be explicitly uncertain. Guidance from the American College of Medical Genetics cautions against using VUS in clinical care, though this is often difficult to adhere to in practice [22,23]. However, here we conceptualize the uncertainty of genetic information more broadly, incorporating the vast array of genetic diagnoses about which little is known and genetic findings that may correlate poorly with a specific neonate’s clinical presentation. In situations where results carry diagnostic and prognostic certainty, their meaning within a clinical and familial context may be less certain [22,24]. There is no straightforward guidance on how such findings should be applied. Importantly, outside of the context of a diagnosis that reveals imminent mortality, results should not be used by clinicians to limit medical interventions that are offered or deemed appropriate [23]. Often, information obtained from genomic testing informs long-term prognoses, such as survival or neurodevelopmental impairment, and may not always influence acute treatment decisions. In these circumstances, when treatment decisions lie within the zone of parental discretion, results require the integration of the infant’s clinical presentation and the family’s values to inform a care path [25]. Clinicians partner with families to understand how the medical information and family perspectives become important in the context of the medical decisions being made.

The authors of this paper are neonatologists, all with additional training in genetics, palliative care, and/or ethics. Using our cumulative training and collective experience, we describe multidisciplinary collaboration in the use of genetic testing results for critically ill neonates, focusing on the challenges posed by uncertain genetic information in circumstances where decisions about ongoing intensive care and the best interests of an infant necessitate that families navigate goals of care. We describe the hypothetical case of an infant with congenital hypotonia and respiratory failure, and consider potential uncertainties and challenges for the family if the case were to proceed with three different genetic testing results: (1) testing reveals a diagnosis which predicts severe chronic respiratory failure and profound developmental disability; (2) testing reveals a variant not previously reported in neonates but associated with moderate impairment when it presents later in childhood; or (3) testing is negative and does not reveal a potential etiology for the clinical picture. We discuss the unique roles neonatologists, geneticists, and palliative care physicians play in supporting the family through decision-making about the best care path. We propose that there should be a structured, collaborative process among subspecialties in order to improve discussions about both indications and the potential utility of genetic testing and the sharing of results with families. Finally, we offer a framework that can be practically applied by interdisciplinary teams to guide parents towards the larger “PICTURE” of genomic testing in neonatal critical care.

## 2. Genomic Testing in the NICU

Each year in the United States >100,000 pregnancies are complicated by a fetus with severe abnormalities, and many neonates are born with congenital anomalies or genetic diagnoses [3,4,5,6]. These diagnoses carry prognoses spanning expectation for survival without significant morbidity, uncertain outcomes, expectation for survival with significant morbidity, or expectation for early mortality [7,8,9,10]. Specifically, studies have shown a high prevalence of genetic disease in critically ill newborns [5,6,12,13,14,15]. Up to 25% of NICU patients have an undiagnosed genetic disorder, and genetic disorders account for 30–40% of morbidity and mortality in the NICU [16,26,27,28,29]. The American College of Medical Genetics strongly recommends that whole-exome sequencing/whole-genome sequencing be considered for neonates with congenital anomalies, as they offer a high diagnostic yield and aid in the timely identification of genetic diagnoses [16,28,30,31]. Early recognition can aid in medical management, improve outcomes, inform counseling, and guide goals of care [26,27]. At times, there is a need to make urgent decisions regarding life-sustaining therapies, and genetic testing results may factor into those decisions [1,2]. Other times, the information provides prognostic information for the family and medical team as they explore different care options [16,17,18]. As whole exome/genome results are returning more quickly, at times within a week, they are increasingly integrated into acute care decisions [1,32]. Importantly, the results should rarely be used to limit medical interventions, as it is deemed appropriate to offer them [23].

While true, a serious genetic diagnosis significantly changes clinical management roughly 65–80% of the time [1,29,32,33]. These changes could include the use of targeted therapies to increase survival, pursuing continued intensive care with a greater understanding of the long-term implications, or transitioning to comfort measures and withdrawing intensive care therapies if a serious or life-limiting condition is identified [1,16,32,33]. Genetic testing results, though, may not always provide more precision or tangible answers for clinicians and families. Testing results may be negative. In these situations, the clinical circumstances have not changed, and there is no further information that may provide reassurance or lead to frustration. In 10–25% of patients, testing may reveal a VUS, a poorly categorized diagnosis, or a diagnosis with a range of potential outcomes [20,21,31,34,35,36]. With a range of possible outcomes, providers may use the additional clinical information to narrow the expected disease trajectory and prognosis. Still, some degree of uncertainty remains, and, at times, providers impact the decision made based on their interpretation of uncertain results [23,36,37]. When the diagnosis is confirmed, there may be a range of phenotypic possibilities and prognoses with no change in the management plan [31,34,38]. Even when the genetic diagnosis and prognosis are definitive, clinicians or parents may process the complex information differently, yielding additional types of uncertainty [22,24]. This is largely because information generated by modern genetic testing is prognostic [25]. Rather than improving medical outcomes in straightforward ways, the value of prognostic information rests on the worth of the information to the families that receive it [25]. Given that the prognosis often signifies disability, or potential disability, an individual’s perception of disability impacts the meaning of the information and creates room for bias and misappropriation in application [18,20,22,24,39,40].

Given all of this complexity, genetic information must be managed carefully to integrate it with family values and maximize benefit [1,41]. Most parents perceive genomic testing as beneficial and have not experienced harm or decisional regret for pursuing it [41,42,43,44,45]. However, there are times when genetic information can be misunderstood, discussed with bias, or inappropriately used to limit treatment options [18,20,22,24,39,40]. In these circumstances, a subset of parents experience stress and concern about the utility of testing [41,43,44]. This necessitates that providers have a deeper understanding of the meaning of all testing results and have an empathic approach to their communication and support [46].

## 3. The Zone of Parental Discretion and the Decision-Making Process

Genetic information must be integrated into the larger context of difficult decision-making in the NICU. As a first step, the neonatologist must determine whether there is more than one medically reasonable option [47,48,49]. Genetic information can be a factor in this determination. For example, if a patient is already known to be critically ill or has significant medical complexity, a genetic diagnosis heralding a poor prognosis may expand reasonable decisions to encompass the possibility of palliative care. Conversely, a genetic diagnosis that heralds a favorable prognosis may tip the scales in the opposite direction, making intensive care obligatory [48]. However, in most cases, a neonate’s phenotype remains the primary determinant of permissible care options, given the wide spectrum of phenotypes associated with many genetic diseases [50,51].

At times, families may wish to pursue options that clinicians deem to fall outside the zone of parental discretion. Genetic information can, at times, fuel disagreements. The parent may believe, rightly or wrongly, that a doctor is overweighting a genetic diagnosis when considering a treatment option. This has occurred most notably for infants with Trisomy 13 and Trisomy 18, where parent narratives highlight potential physician bias when discussing the diagnosis and possible treatment options, and may fail to recognize that some infants survive with varying degrees of medical interventions, while others present with a constellation of findings and congenital anomalies that are non-survivable [52,53,54,55]. In such cases, doctors must be clear and compassionate in their communication, while focusing decisions within the zone of parental decision-making [47,48,56,57]. Parents should be supported and empowered to have wide, but not unlimited, discretion in complex medical decision-making for their children [48,49]. If a dispute arises between parents and clinicians regarding a plan of care where clinicians judge that the parental choice may lead to significant risk of harm versus alternative options that are not overly burdensome and have reasonable chance of benefit, every attempt should be made to understand and resolve the dispute while still protecting the child—including ongoing efforts at communication, consultation with hospital ethics teams, and even outreach to child protective services and/or the legal system if necessary [49].

When there is not a single care option that has a clearly favorable benefit–burden ratio and instead multiple decisions fall within the zone of parental discretion, the clinician should integrate genetic information into shared decision-making. All ethically permissible options should be explained to parents. Here, these options may include the following care plans: comfort measures only, a trial of interventions (which may be tailored to meet specific goals), or full intensive care interventions with the goal of extending the neonate’s life [47,48,58]. To determine which care plan may be most appropriate, clinicians and parents often partner in the collaborative process of shared decision-making [47,59]. Parents rely on clinicians to openly and honestly communicate the diagnosis and prognosis, acknowledging uncertainty and any range of possibilities while eliciting parental hopes, fears, and views on what defines an acceptable quality of life [60]. Clinicians must prepare the family for the decisions that need to be made and help them understand how to interpret that information within their views and values. The decisions that evolve often depend on the certainty of the medical information and the certainty of the family’s value-driven goals.

**Figure 1:**
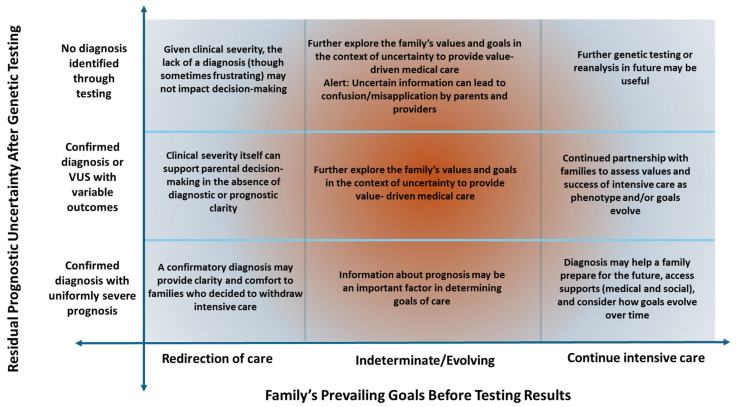
This Figure illustrates a proposed process of parental decision-making depending on the certainty of genetic diagnosis and their goals for a critically ill child. The framework hinges on medical teams having determined which options are within the zone of parental discretion.

We propose that when care decisions are determined to fall within the zone of parental discretion, the impact and utility of modern genetic testing on complex medical decision-making in NICUs is a function of the meaning and certainty of the information itself, as well as how it integrates with a family’s goals of care (Figure 1). Integrating the information into clinical care is challenging, and if the uncertainty within the results is not appropriately addressed, it may engender confusion among clinicians and families as much as it offers guidance [43,61].

If the prognosis is clearly understood and the family is clear in their goals, the information may provide them comfort and allow them to prepare. On the other hand, when the results are negative, it may not impact decision-making. With uncertainty, decision-making can be far more complex.

When clinicians understand the decision-making process and help families learn how to put the medical information in the context of the care of their child, it facilitates bonding, parenting opportunities, and satisfaction with care. More so, it ensures seamless communication, that the wishes of the family are understood, that the family is prepared, that the family has appropriate expectations, and that value-driven medical care is provided [62]. The goals and wishes of the family may shift based on the clinical condition, information received, and parents’ evolving views of what the diagnosis and prognosis mean for their child. This is an iterative process that evolves over time [63,64].

## 4. Multidisciplinary Collaboration

When interpreting genetic testing results, insufficient understanding or inadequate clinical support can lead to the application of genetic information in ways that reflect misunderstanding or bias [18,20,22,24,39,40]. Navigating the complex information generated by modern genetic testing is a multipronged process that requires multidisciplinary collaboration. Neonatologists, geneticists (including medical geneticists and genetic counselors), and palliative care physicians play complementary roles in understanding results and counseling families. Each discipline offers unique training and experiences essential to this context. Broadly, geneticists are experts in understanding genetic information, contextualizing uncertainty, and providing pre- and post-test counseling about results. Neonatologists coordinate complex clinical care plans and are accustomed to assessing and discussing prognosis in an acute care setting. Palliative care physicians specialize in integrating medical information with family values and supporting families through difficult decisions. While interdisciplinary collaboration is essential to caring for complex neonates with many types of disease, the expansion of genetic testing provides a particular collaborative opportunity.

Although collaboration in this context is essential, it can be challenging and impeded by poor coordination or communication amongst specialties. Neonatologists report sending genetic testing frequently, but often without direct support from geneticists [65]. There may be variable access to rapid genomic sequencing across NICUs, impacting the ability to obtain information within the timeframe necessary for decision-making about intensive care therapies [66]. Once the information has resulted, variable availability of subspecialty services and consultation practices by neonatologists for genetic specialists and palliative care teams may limit timely and direct collaboration at the bedside [65,66,67]. There is increased awareness of the growing need for support of families in the setting of genetic testing and interest in advancing collaboration. A recent study exploring perspectives of neonatologists and palliative care clinicians found high agreement between the two groups regarding the need for palliative consultation for patients with significant genetic diagnoses [67].

## 5. Illustrative Case

Baby “Adam” is a 23-day-old infant, born at term gestation after a pregnancy that was notable for polyhydramnios shortly before delivery. He is his parents’ first child and was conceived via conventional in vitro fertilization with autologous gametes. At delivery, Adam is found to have diffuse hypotonia. He is intubated for respiratory failure and transferred to a Level IV NICU within his first week of life. Pediatric neurology is concerned about an underlying neuromuscular diagnosis based on clinical exam, but they are unable to render a clinical diagnosis. Neuroimaging, a newborn screen, and a microarray are non-diagnostic. Adam is now several weeks old and has failed several attempts at extubation, despite being maintained on relatively low ventilator settings. Physicians caring for Adam agree that since Adam’s clinical phenotype is severe and unlikely to improve, it could be ethically permissible for Adam’s family to decide whether to continue or forego mechanical ventilation. Adam’s family is considering tracheostomy for chronic mechanical ventilation. They were counseled extensively about the potential clinical and personal utility of further genetic testing and the range of potential testing outcomes for available testing strategies. They choose to pursue rapid genome testing in hopes of better understanding his neuromuscular condition.

### 5.1. Outcome 1: Results Reveal a Diagnosis with a Clear Prognosis

Adam’s genome sequencing reveals biallelic pathogenic variants in *TBCD.* This variant is associated with severe, early-onset epileptic encephalopathy, for which there is no precise treatment or cure [68,69]. The genetics team counsels the family that the result explains Adam’s symptoms, and that his muscle tone and respiratory status are not expected to improve. Infants with this condition have severe neurodevelopmental impairment, are non-verbal and non-ambulatory, and have progressive seizures [70]. Because this is a recessive condition and both parents are carriers, each future pregnancy has a 25% chance of being affected. Hearing this information, Adam’s parents expressed feeling devastated and emotionally numb. They spend less time in the NICU and defer further conversations with clinicians.

While devastating, the certainty of the genetic findings provides a roadmap for parents and clinicians. Adam’s parents’ reaction likely reflects anticipatory grief, an expected reaction to the serious news. Neonatologists and palliative care providers are both accustomed to accompanying parents through their grief journey. Adam’s parents may need time to process the information before continuing conversations with clinicians. Ultimately, participating in decision-making and planning for Adam can help his parents find a sense of control in this difficult situation: enhancing their coping through honoring their choices [71].

NICU parents report preferring diverse approaches to difficult decision-making, with no single model for these conversations [72]. Personalized dialog promotes autonomy and informed decision-making. The focus should not merely be on the information that clinicians want to convey. True shared decision-making should center upon eliciting Adam’s family’s goals and values, incorporating these into the medical decisions [11]. Of particular importance to this case, families hold diverse ideas about disability and caring for children with disabilities [73]. Clinicians in a collaborative approach can best bring forth such ideas and effectively provide recommendations for Adam’s care that align with his family’s wishes. For outcome 1, neonatologists and palliative care clinicians face challenges that are not unusual in the NICU, as many diagnoses unfortunately portend long-term, severe problems with health and development.

### 5.2. Outcome 2: Results Reveal a Diagnosis with an Uncertain Prognosis

Adam’s testing reveals a de novo deletion in *PAX5*, a transcription factor linked to a neurodevelopmental disorder characterized by delayed developmental milestones, intellectual disability, and/or autism spectrum disorder [74]. Though the variant has been reported as pathogenic in older children, it has not been previously identified as causative for neonatal symptoms. Adam’s parents express confusion about the results and feel the information has further complicated their decision about a tracheostomy.

The uncertainty of this finding, specifically, its significance in a symptomatic neonate, creates challenges for Adam’s parents and doctors. The variable interpretation and use of such incidental findings pose many challenges. Having a named diagnosis will help some families feel comfortable making decisions about a care trajectory despite the current gaps in understanding. For others, this additional information in the context of unclear implications may further obscure decisions. Genetic information must be managed carefully to ensure that it is integrated with family values to maximize benefit [1,41]. As a first step, parents need to understand the implications of the diagnosis and the residual uncertainty. Then, parents need support in determining how this uncertain information factors into their decisions, a realm in which palliative care physicians have the greatest expertise [59,75,76,77]. While most parents perceive genomic testing as beneficial, when there is insufficient preparation for, or understanding of, results, parents can express concern, like Adam’s parents do [18,20,22,24,39,40,41,42,43,44,45]. Providers must have a complete understanding of all testing results and their implications while offering empathetic guidance and support [44,46].

### 5.3. Outcome 3: Results Reveal No Pathologic Variant

Adam’s genomic sequencing results are negative, not revealing an etiology for his symptoms. These results are conveyed during a care conference with genetics, neonatology, and palliative care. Adam’s parents immediately express frustration, stating, “This testing was supposed to give us answers. Now what are we supposed to do?”

Though a negative genome adds diagnostic value by eliminating potential etiologies, Adam’s family expresses that they do not have “new” information for decision-making. His parents had been counseled prior to testing that negative results were a possibility. However, even the most thorough pre-test counseling may not fully convey this message. Stressed NICU parents, worried about their child’s future, may have difficulty processing complex information. Analogous to the phenomenon of “therapeutic misconception” reported amongst participants in phase 1 clinical trials, families who choose to proceed with genomic testing may do so believing that they personally will receive definitive information or benefit from pursuing testing [43,78].

A non-diagnostic genomic result threatens to inappropriately impact medical decision-making by allowing for interpretation through the lens of individual bias. A non-diagnostic result could make a genetic etiology for Adam’s symptoms seem less likely, though the extent to which this is true is disease-dependent [79]. Whether accurate or not, this could be interpreted as a positive indicator of long-term recovery, spurring Adam’s family and team to proceed with tracheostomy. Absence of a diagnosis could alternatively be interpreted as rendering Adam’s condition a “medical mystery,” leading his medical team and family to feel they are unlikely to find a path towards his long-term survival and thus plan for withdrawal of mechanical ventilation. Negative results may also engender mistrust. Adam’s parents, feeling they were promised more precise answers from genomic testing, may feel “stuck” without that precision. This may delay a decision on tracheostomy, as the family searches for additional information from repeated imaging or second opinions.

## 6. Discussion: Towards Developing a Collaborative Framework

Given its impact on outcomes, length of stay, targeted therapy, and cost of care, genomic testing is becoming increasingly prevalent early in the NICU course for critically ill neonates like Adam [25,80,81,82,83,84,85,86]. However, incorporating results into decision-making in meaningful ways is not always straightforward. Genetic results pose challenges in both understanding the results and meaningfully applying them to medical decisions [43,61]. The impact and utility of modern genetic testing is a multifaceted interplay of the clinical context, standards of medical care, timely results, accurate interpretation of results, and communication of the results.

The American Academy of Pediatrics supports shared decision-making through multiple conceptual models that emphasize the interplay of clinician guidance and parental involvement that depends on the clinical context, prognostic certainty, treatment options, and family values [47,48,49,59]. One such model provides a 4-step framework to determine when and how to implement shared decision-making between a clinician and parents [47]. This framework offers guidance to address clinical scenarios with a wide range of disease states. Step 1 addresses the medical reasonableness of one or multiple medical options. Step 2 is a benefit–burden comparison. If one treatment option has a clear benefit–burden option on clinical grounds, this would support a physician-guided approach that incorporates family values and preferences. Step 3 incorporates variation in what matters most to families based on their personal values. If parents have high preference sensitivities to physician-guided recommendations, parental involvement will be of greater importance. Step 4 is calibrating the shared decision-making between physician-guided shared decision-making and parent-guided shared decision-making. This 4th step may change over time based on the evolution of the medical condition and response to therapy.

We propose that in situations where decisions about care paths are within the zone of parental discretion, the impact and utility of complex medical decision-making in NICUs are a function of the meaning and certainty of the information itself, as well as how it integrates with a family’s goals of care (Figure 1).

Clinicians who do not sufficiently understand genetic testing results or the role of results in the clinical context may provide insufficient or inappropriate counseling. For instance, a geneticist could tell the family of an infant with multiple life-threatening anomalies that survival is not limited by a specific genetic diagnosis if they are solely relying upon the literature reports that describe mild phenotypes. A neonatologist could tell the parents of an infant with trisomy 18 that the condition is expected to be lethal soon after birth, even if the infant has no life-limiting anomalies, if they are not up to date on the more recent literature about outcomes for the diagnosis [54]. Instead, they should realize there is a spectrum of outcomes that, at times, largely depend on patient-specific factors and the goals of care. Optimal prognostication requires interprofessional cooperation.

**Figure 2:**
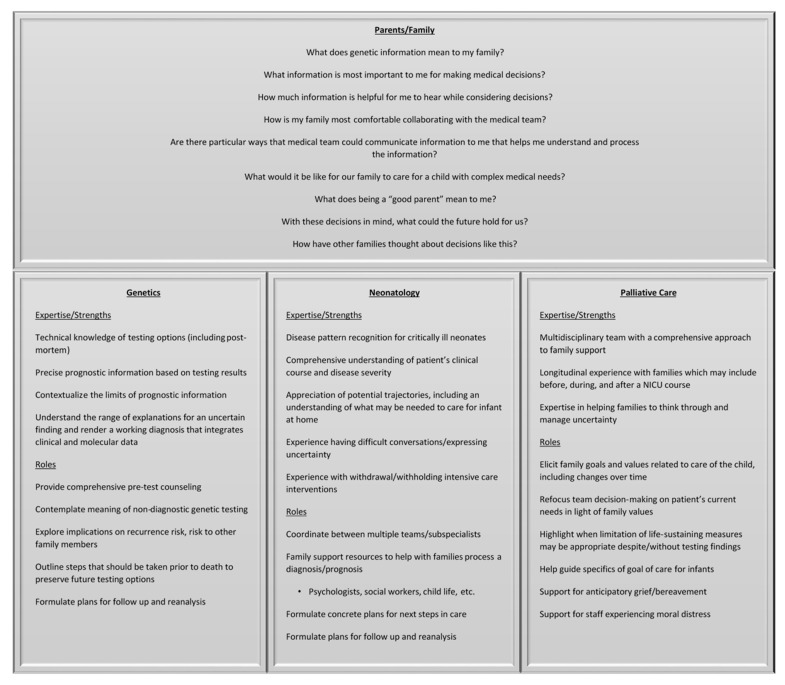
This Figure illustrates how each subspecialty contributes to supporting the family on their journey as they process the information and determine the care path that is best for their child.

In each outcome for our case, Adam’s family navigated a unique variety of uncertainty while making future-facing decisions for their son following genomic testing (Figure 1). Uncertainty is inherent in all diagnostic tests, and yet, uncertainty in genetic testing carries greater weight, because of the perceived superiority of genetic testing in ‘solving the puzzle,’ the novelty of the tests, and the implications for management beyond the patient (e.g., reproductive testing) [87,88,89,90]. Modern genetic testing inherently involves uncertainty, particularly about prognosis. This should be expected, understood, and conveyed to families as an inherent feature of the test result as a whole, rather than as something arising from the laboratory classification of a specific variant as a VUS. We posit that families, especially those with evolving or uncertain goals of care, will be best supported through this expected uncertainty with balanced counseling through collaboration between the family and clinicians from neonatology, genetics, and palliative care (Figure 2).

Our approach is geared to a specific subset of neonates with a high likelihood of genetic abnormalities, including conditions associated with a clearly poor prognosis or genetic variants of uncertain significance. Since these scenarios occur relatively commonly in neonatal intensive care, it would benefit clinicians to develop a focused collaborative process that integrates critical care management (neonatology), diagnostic/prognostic expertise (genetics), and longitudinal, goal-oriented considerations (palliative care). A consistent multidisciplinary approach facilitates a shared understanding between clinicians and families, supports alignment of medical recommendations with family values, and allows for recalibration of decision-making as the clinical course and diagnostic clarity evolve.

Parents should be considered experts on their children, their goals, and their values. For families without experience with serious medical decision-making, it may be difficult to articulate what could be helpful to them through the process. Families have different preferences for information sharing: what is conveyed, how it is communicated, and by whom it is shared. Each parent has a unique view of what it means to be a “good parent,” which can strongly influence decisions [91,92]. Families may differently conceptualize what it would mean to care for a child with complex medical needs and the impact of medical complexity on their child’s quality of life. Clinicians who accompany families on such diagnostic journeys should be prepared to guide them through such considerations.

It has become routine for neonatal and genetic clinicians to have pre-test discussions with families prior to genetic testing. Palliative care consultation should become standard prior to testing when decisions regarding life-sustaining therapies are being considered. Pre-test counseling should facilitate informed decision-making about testing for the patient, incorporating family goals and values [93]. These discussions should include reasons for testing, range of possible results and turnaround time, limitations of genetic testing, potential benefits versus harms of testing, and the cost of testing [1]. Exploring how potential results may influence stakeholders and how they anticipate using the information (treatment, understanding the prognosis, and determining goals of care) ahead of time is important [94]. Comprehensive pre-test counseling can help families prepare for possible outcomes and facilitate discussions about values and views. Some families may decide that the outcome predicted by a child’s clinical course, irrespective of testing results, does not support an acceptable quality of life for them. This value judgment should be incorporated by the medical team when identifying potential and/or recommended testing strategies. Options include declining testing, pursuing testing options likely to result after an anticipated patient demise, or decoupling the receipt of results from medical decision-making. Importantly, the involved medical teams should anticipate that the range of reactions evidenced in the vignettes above may manifest despite competent pre-test genetic counseling.

Each medical subspecialty has unique training and skills that, in concert, can provide a strong foundation to support families through making decisions with uncertain information (Figure 2). Geneticists have access to the most complete, up-to-date genotype-driven prognostic information and can contextualize the limits of that data for families. Geneticists consider all possible explanations for an uncertain finding and render a working diagnosis that integrates clinical and molecular data. This includes considering the limitations of testing modalities. When a genetic diagnosis is made, geneticists can provide guidance about recurrence risk for future pregnancies as well as implications for other family members. If a patient were to decompensate prior to genetic testing, geneticists can discuss post-mortem testing options and steps that should be taken prior to death to preserve future testing options. Finally, geneticists can develop post-testing follow-up plans, including reanalysis of previously non-diagnostic findings.

As the primary medical specialists for critically ill infants, neonatologists have the most comprehensive sense of an individual patient’s clinical course and disease severity. They are accustomed to incorporating non-specific test results into the context of clinical situations to tailor treatment plans based on the collective information. Using knowledge of the information available and likely disease trajectories, neonatologists can guide families through what a condition may mean for a child, both in the short and long term. This may include surgical procedures, such as tracheostomy and gastrostomy placement, to enable the use of medical technology for eventual discharge home. As intensivists, neonatologists are experienced in having difficult conversations with families, including the withholding or withdrawal of life-sustaining therapies. Recognizing that NICU admissions are experienced by families as trauma, many NICUs employ multidisciplinary teams of professionals (including nurses, social workers, psychologists, child life specialists, and others) to provide support through difficult journeys.

Interdisciplinary palliative care teams add additional resources, with comprehensive approaches to family support. Many palliative care teams have longitudinal relationships with families and assist with medical decision-making beyond the NICU. With the time and expertise to engage families in talking about difficult subjects, palliative care clinicians can elucidate a family’s most closely held values and then center discussions on the patient’s current medical needs to promote those. Overlap between disciplines will occur and should be considered a strength, as it fosters balanced counseling [67].

Collaboration necessitates appreciation of unique strengths and willingness to work collectively, as well as structures to make information and idea-sharing feasible. The teams should come together during two broad time points: when testing options are discussed and when results are reviewed. Genetic consultation for early, broad genomic testing should be considered when a patient’s care could be improved by either a molecular diagnosis or elimination of potential etiologies through non-diagnostic testing [95]. A growing body of literature demonstrates the benefit of early palliative care consultation for pediatric patients [96]. We recommend that when the goals of care are not clearly established, and genetic testing is being contemplated, neonatologists consult genetics and palliative care teams to assist in deliberations. Testing should be framed as a choice for families to avoid adding to the “clinical momentum”, which has been reported to complicate intensive care [97]. Subspecialists need to reunite once results are available to carefully consider the meaning of genetic information in the context of a patient’s clinical status and what is known about a family’s values in order to determine how best to communicate the test results. Representatives from each subspecialty should jointly disclose results to the family, each adding their perspective and support. Expecting families may need time and space to consider challenging information; the teams should debrief after the disclosure and plan to coordinate ongoing follow-up. This comprehensive multidisciplinary approach to care may pose challenges in NICU settings without subspecialty availability. This, though, does not negate the importance of the care model. Hospitals can establish an integrated care network or telehealth consultation to incorporate a multidisciplinary team into the evaluation and counseling process.

As a framework to guide this recommended subspecialty collaboration, we posit that clinicians focus on concrete ways to help families see the whole “PICTURE” of genomic testing in neonatal critical care. We developed the novel PICTURE mnemonic based on expert opinion to encapsulate and highlight key components to the collaborative process: **P**reparation, **I**nterdisciplinary work, **C**onsistency, **T**ransparency, **U**ncertainty acknowledgement, **R**ecognizing limitations, and **E**ngagement. Table 1 lists the PICTURE components and presents key considerations for each component. Neonatal, genetic, and palliative care teams working to build or develop collaborative systems of practice should reflect on how the PICTURE framework can be adapted to suit their collective needs.

## 7. Conclusions

As genetic testing for critically ill infants continues to evolve, so must the ways that subspecialists and families collaboratively care for these infants. Diagnostic and prognostic information should be shared in ways that encourage families to process information, ask questions, and use the information to make empowered decisions. To provide equitable care for neonates with genetic variants, there is an ethical imperative to study communication about testing and results, including the proposed collaborative model and PICTURE mnemonic, and its long-term impacts on patients and families [40]. Future research, partnering with patient and family stakeholders, is needed to ensure that diverse perspectives lead the way forward. Currently, limitations include a lack of standardized guidelines for integrating genetic results into goals-of-care discussions and gaps in long-term outcome data. Ongoing research and consensus-building are needed to optimize the use of genomic information in guiding care decisions for critically ill neonates.

## Figures and Tables

**Table 1:** This Table outlines the PICTURE framework to guide families as they begin to understand the whole “picture” of genomic testing in critical care.

**P**	**Prepare** Preparing teams ○ To minimize the impact of collaborative “growing pains” on patients and families ○ Ensure shared mental model in which decisions fall into the zone of parental discretion, and how genetic results may impact these Preparing families ○ Ensure appropriate pre-test counseling happens ○ Provide anticipatory guidance throughout the process of testing ○ Be explicit about how such testing may intersect with medical decision-making and how it may not Emphasize the importance of education for staff and families
**I**	**Interdisciplinary Collaboration** Beyond multidisciplinary: all teams must work closely together Develop a process to streamline communication between the three disciplines
**C**	**Consistency** Standardize the approach to genetic testing and counseling Collaborate regularly ○ Remove subjectivity/bias from decisions about when to consult Use “trigger diagnoses” to standardize palliative care consultation ○ Increases frequency and improves perceptions of value
**T**	**Transparency** Families should clearly understand how we might use the information ○ May influence how we counsel about goals of care ○ May affect eligibility for treatments Clarifying whether family and/or clinical teams’ expectations might change based on information ○ May or may not change the clinical outlook ○ Be as clear as possible ○ Include information on which decisions may fall out of the zone of parental discretion
**U**	**Uncertainty Acknowledgment** Uncertainty will always be part of genetic testing Introducing new practices and collaborations often entails challenges We need formal, validated ways to communicate uncertainty about genetic information, which do not currently exist
**R**	**Recognizing Limitations** Limitations of available testing Limitations of resultant genetic information Limitations of the strengths and knowledge of individual clinicians
**E**	**Engagement** Ongoing engagement of clinicians to refine local processes ○ Goal of sustainability ○ Iterative process Ensure testing and counseling optimally support families ○ Engagement of patient and family stakeholders is critical

## Data Availability

Not applicable.

## Abbreviations

The following abbreviations are used in this manuscript:

NICU	Neonatal intensive care unit
VUS	Variant of unknown significance
